# BrLETM2 Protein Modulates Anthocyanin Accumulation by Promoting ROS Production in Turnip (*Brassica rapa* subsp. *rapa*)

**DOI:** 10.3390/ijms22073538

**Published:** 2021-03-29

**Authors:** Hyon Dok Song, Jianfei Yang, Nam Hyok Mun, Bowei Chen, Yunzhu Chen, Pyol Kim, Saneyuki Kawabata, Yuhua Li, Yu Wang

**Affiliations:** 1 Key Laboratory of Saline-alkali Vegetation Ecology Restoration (Northeast Forestry University), Ministry of Education, Harbin 150040, China; shd@nefu.edu.cn (H.D.S.); yjf19910304@126.com (J.Y.); mnh@nefu.edu.cn (N.H.M.); chenbw@nefu.edu.cn (B.C.); cyzcarol@foxmail.com (Y.C.); kimpyol1119@nefu.edu.cn (P.K.); 2 College of Life Science, Northeast Forestry University, Harbin 150040, China; 3 Korean Horticultural Association, Pyongyang 999093, Democratic People’s Republic of Korea; 4 Institute for Sustainable Agroecosystem Services, Graduate School of Agricultural and Life Sciences, The University of Tokyo, Midoricho, Nishitokyo, Tokyo 188-0002, Japan; skawabata@g.ecc.u-tokyo.ac.jp

**Keywords:** anthocyanin, bulk segregant analysis, BrLETM2, ROS

## Abstract

In ‘Tsuda’ turnip, the swollen root peel accumulates anthocyanin pigments in a light-dependent manner, but the mechanism is unclear. Here, mutant *g120w* which accumulated extremely low levels of anthocyanin after light exposure was identified. Segregation analysis showed that the anthocyanin-deficient phenotype was controlled by a single recessive gene. By using bulked-segregant analysis sequencing and CAPS marker-based genetic mapping analyses, a 21.6-kb region on chromosome A07 was mapped, in which a calcium-binding EF hand family protein named BrLETM2 was identified as the causal gene. RNA sequencing analysis showed that differentially expressed genes (DEGs) between wild type and *g120w* in light-exposed swollen root peels were enriched in anthocyanin biosynthetic process and reactive oxygen species (ROS) biosynthetic process GO term. Furthermore, nitroblue tetrazolium (NBT) staining showed that the ROS level decreased in *g120w* mutant. Anthocyanins induced by UV-A were abolished by the pre-treatment of seedlings with DPI (an inhibitor of nicotinamide adenine nucleoside phosphorylase (NADPH) oxidase) and decreased in *g120w* mutant. These results indicate that BrLETM2 modulates ROS signaling to promote anthocyanin accumulation in turnip under UV-A and provides new insight into the mechanism of how ROS and light regulate anthocyanin production.

## 1. Introduction

Anthocyanins are polyphenolic secondary metabolites that are ubiquitous in the plant kingdom. They play a role not only in reproduction, by attracting pollinators and seed dispersers, but also in protection against various abiotic and biotic stresses [1]. For consumers, anthocyanin-enriched fruits and vegetables are considered to be healthy and delectable, which are important target traits in horticulture plants breeding programs.

Anthocyanins are produced from a well-characterized pathway that contains key structural genes: CHALCONE SYNTHASE (CHS), CHALCONE ISOMERASE (CHI), FLAVANONE 3-HYDROXYLASE (F3H), DIHYDROFLAVONOL-4-REDUCTASE (DFR), ANTHOCYANIDIN SYNTHASE (ANS), and UDP-GLUCOSE: FLAVONOID 3-O-GLUCOSYLTRANSFERASE (UFGT). The regulation of anthocyanin biosynthesis pathway is mediated by the regulatory MBW complex, in which R2R3-MYB, basic helix-loop-helix (bHLH), and WD40-repeat (WDR) transcription factors (TFs) are involved [2]. In Arabidopsis, the key components of the complex—R2R3-MYB protein PRODUCTION OF ANTHOCYANIN PIGMENT1 (PAP1) and bHLH protein TRANSPARENT TESTA 8 (TT8)—are able to directly bind the promoter region of anthocyanin biosynthetic genes such as DFR and ANS and activate their expression [3,4,5]. Both R2R3-MYB and bHLH TFs in many species are regulated by various environmental stimuli to induce anthocyanin biosynthesis in a temporal and spatial manner [6,7,8].

Light is one of the most important environmental inducers of anthocyanin accumulation [9,10]. The regulation mechanism of the MBW complex by light is complicated. Light suppresses a negative regulator CONSTITUTIVE PHOTOMORPHOGENIC1 (COP1) to stabilize light-responsive factors, including ELONGATED HYPOCOTYL 5 (HY5), B-box proteins (BBXs), PAP1 and PAP2, for upregulating anthocyanin biosynthesis [11,12]. On the other hand, HY5 and BBXs are thought to be involved in the light-induced anthocyanin pathway by activating the R2R3-MYB regulators and biosynthetic genes of anthocyanin pathways [13,14]. Moreover, the reactive oxygen species (ROS) is reported to participate in light-induced anthocyanin accumulation [15,16,17]. Studies of ROS inhibitor diphenylene-iodonium chloride (DPI) and ROS inducer paraquat have revealed that the repression of NADP oxidase activity leads to decreased anthocyanin levels, while induction of ROS generation triggers anthocyanin accumulation by activating the regulatory genes *PAP1* and *TT8* in *Arabidopsis* [10,18]. Anthocyanin-deficient mutants had relatively higher ROS levels than wild type after paraquat treatment, indicating that anthocyanins function as antioxidants to protect against ROS in plants. Transcriptome analysis of seedlings grown in the dark, light, or treated with H_2_O_2_ showed that a high percentage of H_2_O_2_-responsive genes are also regulated by light, which suggests that light induces H_2_O_2_ signaling in *Arabidopsis* [19]. During de-etiolation, HY5 induces some ROS-responsive gene expression to scavenge singlet oxygen that generated by photosensitized protochlorophyllide under light [20]. Although light is known to affect ROS production, the mechanism by which light regulates ROS production and anthocyanin accumulation is poorly understood.

Turnip (*Brassica rapa* subsp. *rapa*) is one of the most important horticultural vegetables that is widely distributed throughout the world. The swollen root of turnip displays rich morphotypes such as size, shape, and color during domestication. White, green, red, and purple turnip cultivars have been found, which provide enriched nutrition that benefits human health [21,22]. The purple cultivar Tsuda from Japan is enriched in anthocyanins in the peels of its swollen root when exposed to light. Previous studies showed that UV-A irradiation or co-irradiation with blue and UV-B (blue + UV-B) are the most efficient wavelengths for promoting anthocyanins accumulation in the peels of swollen roots of ‘Tsuda’ [23,24]. Transcriptome analysis identified expression patterns of light-responsive genes and anthocyanin biosynthetic genes in response to UV-A and Blue + UV-B, indicating that an overlapped signaling transduction regulatory network may exist to regulate anthocyanin accumulation. Furthermore, ROS-scavenging pathway genes were upregulated after UV-A irradiation [24]. The mechanism of how UV-A regulates anthocyanins of swollen root peel, however, also remains unclear.

Toward understanding how UV-A regulates anthocyanin accumulation in turnip, we previously constructed a T-DNA library by an improved Agrobacterium-mediated vacuum infiltration method [25]. We screened mutants with abnormal anthocyanin traits of swollen root peel in T_2_ generation and identified desirable mutants that displayed a heritable phenotype in T_3_ generation. Among them, we found an anthocyanin-deficient mutant, *g120w*. In the present study, we identified *BrLETM2* as the candidate gene for the mutation in *g120w* using a map-based cloning strategy, combined with a bulked-segregant analysis (BSA) and a modified BSA-seq method. The transcriptome analysis and histochemical staining results indicated that BrLETM2 is involved in light-induced anthocyanin accumulation by promoting ROS levels in turnip.

## 2. Results

### 2.1. Phenotypic Characterization and Genetic Analysis of g120w Mutant

In Tsuda turnip, light-exposed swollen root peel accumulates large amounts of anthocyanins. The *g120w* mutant was identified from a T_2_ population of our previous T-DNA insertion library [25]. However, we failed to amplify fragments of reporter genes of the insertion vector in *g120w*, which may be due to a loss of T-DNA insertion during reproductive generation, but it still displayed a heritable anthocyanin-deficiency phenotype.

**Figure 1:**
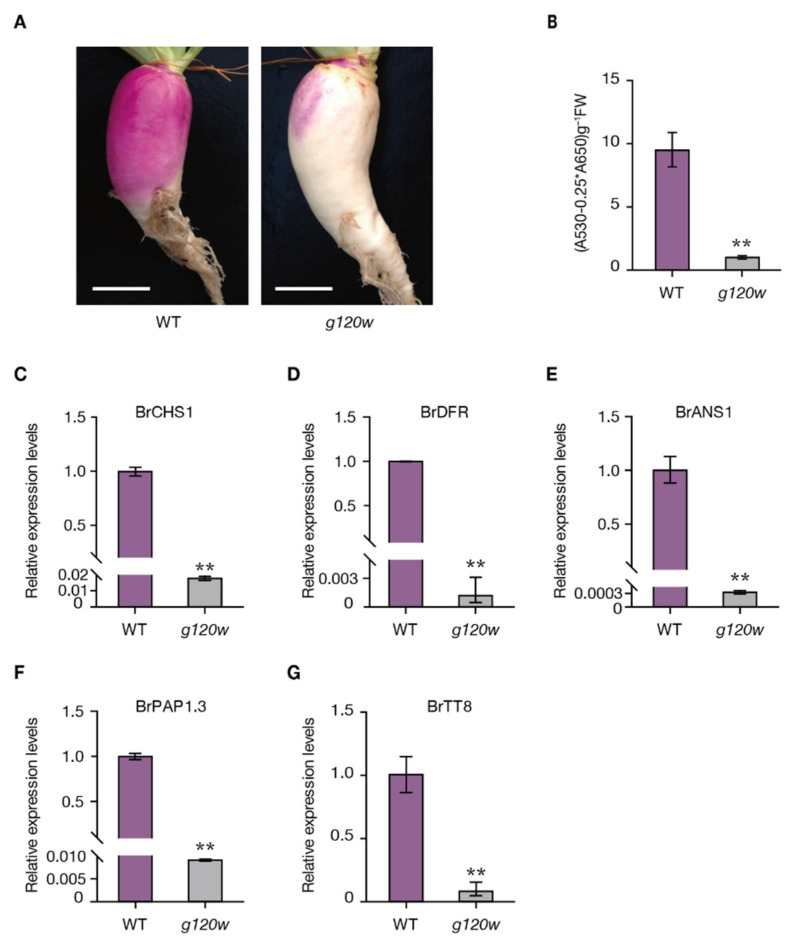
Characterization of *g120w* mutant. (**A**) Phenotypic variation in the swollen root of the wild type and *g120w*. Bars = 5 cm. (**B**) Anthocyanin content in wild type and *g120w* swollen root peel. (**C**–**G**) Quantitative real-time PCR (qRT-PCR) analysis of *BrCHS1*, *BrDFR*, *BrANS1*, *BrPAP1.3*, and *BrTT8* expression levels in swollen root peels in (**A**). Mean (±SD) expression levels were normalized by *BrACTIN7*, and results for wild type were set at 1. Asterisks indicate a significant difference between WT and *g120w* (** *p* < 0.01, Student’s *t*-test, *n* = 3).

When exposed to light, the swollen root peel of *g120w* showed reduced purple pigmentation compared to the wild type (Figure 1A) and measurements of anthocyanin showed that *g120w* had accumulated less anthocyanins in swollen root peel (Figure 1B). Consistent with the anthocyanin-deficiency phenotype, the expression level of anthocyanin-biosynthetic genes such as *BrCHS1*, *BrDFR*, and *BrANS*1, and regulatory factors *BrPAP1.3* and *BrTT8* were robustly decreased in *g120w* (Figure 1C–G).

To characterize the inheritance of *g120w*, we used *g120w* as the maternal plant in a cross with the wild type and generated the F_1_ population, which was self-pollinated to generate the F_2_ population. Phenotypic segregation analysis showed that the F_1_ progeny had a purple peel. For the 270 F_2_ individuals, 204 had purple peels (PP-type) and 66 had white peels (WP-type), which was consistent with the predicted 3:1 segregation (χ^2^ = 0.833). The genetic analysis of *g120w* showed that the anthocyanin deficiency trait was caused by a single recessive gene.

### 2.2. Identification of Candidate Gene by BSA-seq

**Figure 2:**
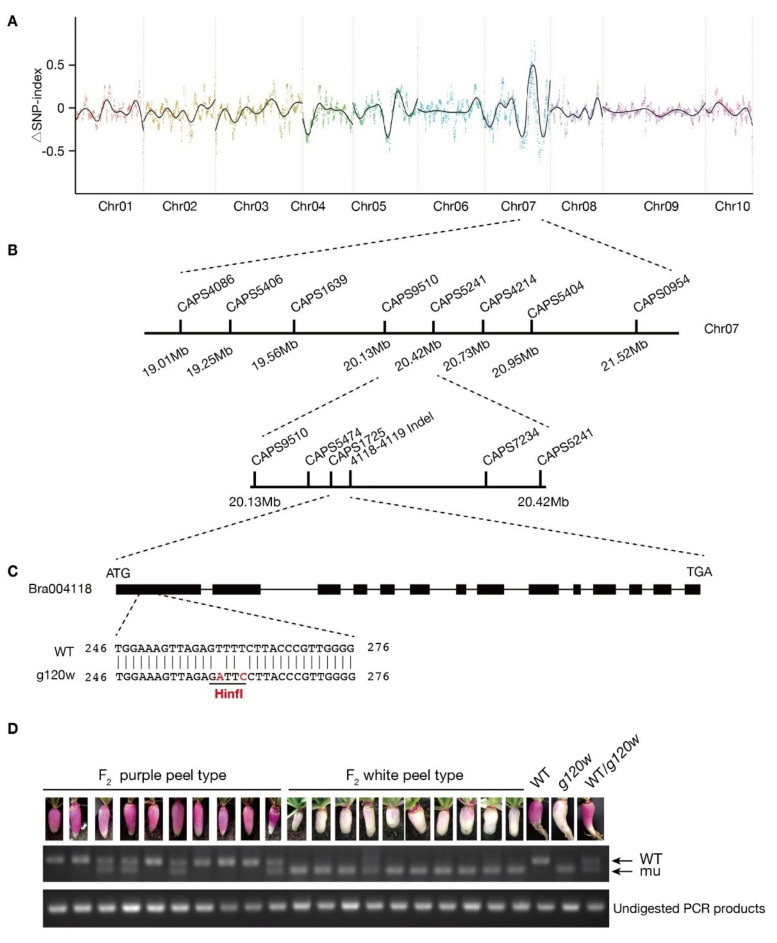
Map-based cloning of *g120w*. (**A**) The calculation of ΔSNP-index values on chromosomes to identify the candidate regions that associated with the anthocyanin deficiency trait between the two segregate bulks of BSA-Seq (PP-type and WP-type). The curved black lines are average Δ(SNP-index) values. *X*-axis, physical position; *Y*-axis, SNP index. (**B**) Genetic mapping of the candidate gene using CAPS markers. (**C**) Diagram of structure of *BrLETM2* gene and amino acid variation of BrLETM2 protein between wild type and *g120w*. Black box indicates exons. (**D**) Genotypic analysis of CAPS1725 in first exon of *BrLETM2*. Two nucleotide variation led to digestion of fragments in *g120w* by restriction enzyme Hinf1. All the detected F_2_ progeny with the wild type or mutant phenotype co-segregated with CAPS1725.

For identifying the gene responsible for the low anthocyanin pigmentation in *g120w*, four DNA libraries, two parental lines (WT, *g120w*) and two bulked F_2_ populations (PP-type and WP-type) generated by the Illumina HiSeqTM2500 platform were used for BSA-seq analysis. A Δ(SNP-index) comparison between DNA pools of the PP-type and WP-type progeny revealed a significantly different pattern on chromosome A07 in the region from 19.00 Mb to 21.86 Mb associated with the *g120w* locus (Figure 2A). In the candidate region, 18 SNPs that caused nonsynonymous variations in the coding region of 15 genes were identified (Table S1).

To narrow the candidate region in the *g120w* locus, 10 CAPS markers and one indel marker within the candidate region were developed for fine mapping (Figure 2B). Five recombinant plants of WP-type F_2_ progenies were used to the narrow the candidate area in *g120w* to a 21.8-kb region, between the markers CAPS5474 and 4119INDEL, in which only contains one candidate gene *Bra004118*. From a functional annotation analysis, Bra004118, which encodes a calcium-binding EF hand family protein, was designated as BrLETM2 since it had a high similarity to AtLETM2 in *Arabidopsis*. To identify variations in *BrLETM2* gene, the full length of *BrLETM2* was cloned from the swollen root peel of wild type and *g120w*. A sequence alignment showed that the BrLETM2 from *g120w* contains a seven-amino-acid variation and one amino acid insertion in the first exon (Figure S1). Then, a CAPS1725 marker was designed to confirm an association between the genotypic variation in BrLETM2 with anthocyanin deficiency. The *BrLETM2* gene in *g120w* had two nucleotide variations compared with wild type, which could be specifically digested by restriction enzyme HinfI in the PCR-amplified fragment of CAPS1725 (Figure 2C). The genotypic assay showed that CAPS1725 co-segregated with the anthocyanin deficiency phenotype in F_2_ population (Figure 2D). Therefore, *BrLETM2* is presumed as the candidate gene in *g120w*.

### 2.3. Spatiotemporal Transcription and Phylogenetic Analyses of BrLETM2

**Figure 3:**
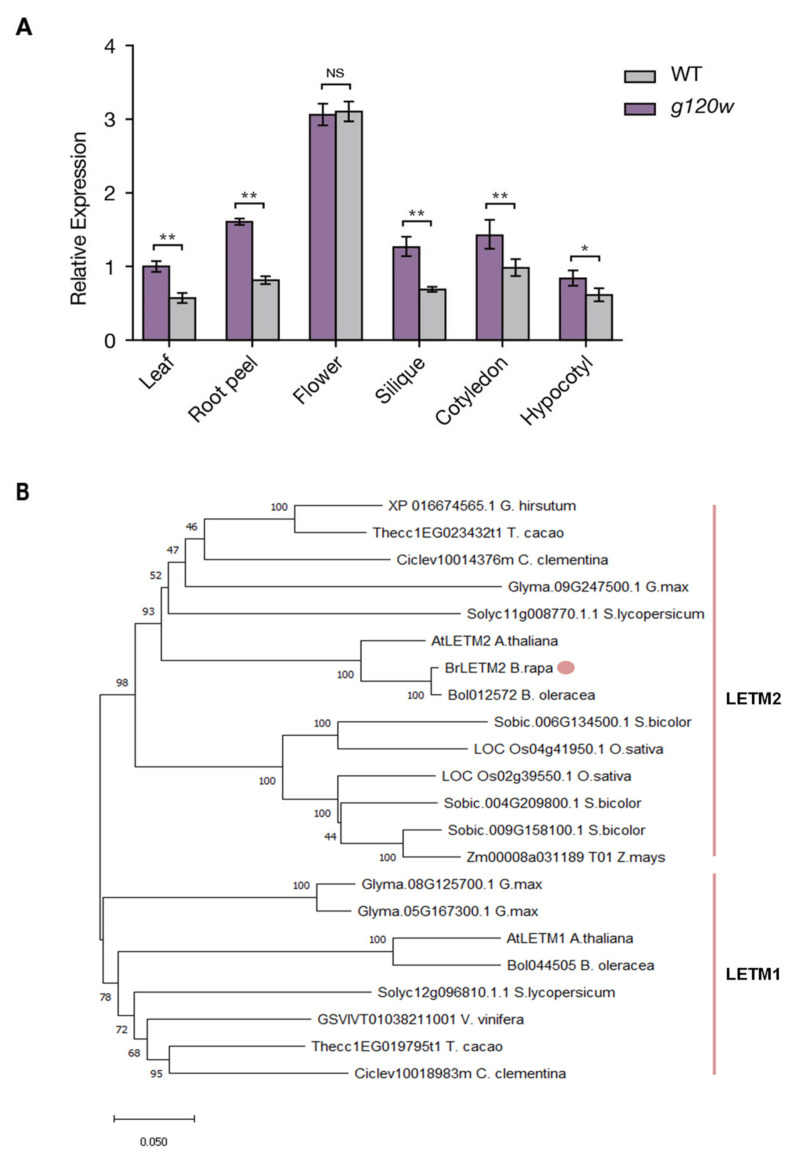
Expression analysis of *BrLETM2* in wild type and phylogenetic tree of LETM2 proteins among different plant species. (**A**) qRT-PCR analysis of *BrLETM2* expression in wild type and *g120w* in different organs. Values are means ± SD (*n* = 3); asterisks indicate a significant difference between wild type and *g120w* among organs by Student’s *t*-test (* *p* < 0.05, ** *p* < 0.01). (**B**) Phylogenetic tree constructed using the neighbor-joining method for LETM protein sequences. The tree is drawn to scale, and branch lengths indicate evolutionary distance.

The expression of *BrLETM2* was then quantified in various organs in wild type and *g120w* using qRT-PCR. In wild type, the highest expression of *BrLETM2* was in the flowers, followed by the root peels, cotyledon, and silique. The lowest *BrLETM2* expression levels were in the leaves and hypocotyls (Figure 3A). Except for flowers, the expression of *BrLETM2* in other organs was lower in *g120w* than those in the wild type. In a phylogenetic tree to elucidate the evolutionary relationships of BrLETM2 with other LETMs orthologs of various species, BrLETM2 clustered with various dicots and was closely related to AtLETM2 (Figure 3B).

### 2.4. Ectopic Expression of the BrLETM2 Promotes Anthocyanin Accumulation in Arabidopsis

**Figure 4:**
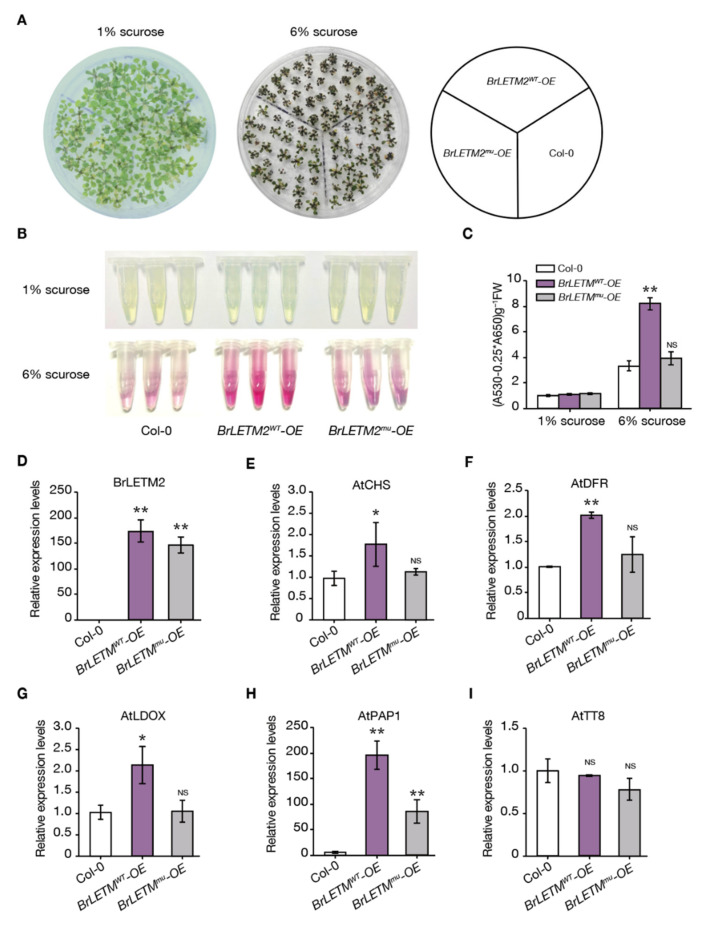
BrLETM2 promotes anthocyanin biosynthesis in response to high sucrose in 14-day-old seedlings of *Arabidopsis*. (**A**) Seedlings of Col-0, *BrLETM2^WT^-OE* and *BrLETM2^mu^-OE* were grown on plates with normal sucrose (1%) and high sucrose (6%). (**B**,**C**) Anthocyanin levels of seedlings in (**A**). (**D**–**I**) Expression levels of *BrLETM2*, *AtCHS*, *AtDFR*, *AtLDOX*, *AtPAP1*, and *AtTT8* in seedlings in (**A**) were quantified by qRT-PCR. Expression level of Col-0 was set at 1 and *AtACTIN8* was used for normalization. Values are means ± SD (*n* = 3); asterisks indicate a significant difference between Col-0 and overexpression lines (* *p* < 0.05, ** *p* < 0.01, Student’s *t*-test).

To verify whether BrLETM2 participates in anthocyanin biosynthesis, the full length of *BrLETM2* from WT and *g120w* were cloned into pCMP1-35S vector and separately inserted into Col-0 *Arabidopsis* using an *Agrobacterium*-mediated flower dipping method. *BrLETM2^WT^-*overexpressing (*OE*) and *BrLETM2^mu^*-*OE* seedlings accumulated extremely low levels of anthocyanin as Col-0 under control condition, while high sucrose-grown seedlings displayed a dwarf and enriched anthocyanin phenotype (Figure 4A). The *BrLETM2^WT^*-*OE* seedlings accumulated more anthocyanins than the wild type and *BrLETM2^mu^-OE* seedlings when treated with high sucrose (Figure 4B,C). The mRNA of *BrLETM2* was significantly induced at a comparable level in *BrLETM2^WT^-OE* and *BrLETM2^mu^-OE* seedlings (Figure 4D). The anthocyanin biosynthetic genes *AtCHS*, *AtDFR*, *AtLDOX*, and *AtPAP1* were more highly expressed in *BrLETM2^WT^-OE* seedlings than those of in *BrLETM2^mu^-OE* and wild type, but not *AtTT8* (Figure 4E–I).

### 2.5. RNA-seq Analysis Revealed Enrichment of the Anthocyanin and ROS Signaling Pathway in g120w

To understand how BrLETM2 regulates anthocyanin biosynthesis, RNAs from light-exposed swollen root peel of wild type and *g120w* were sequenced and analyzed. Deep sequencing yielded 59 to 75 million reads from the wild type and *g120w* samples. After quality control, 84% of the reads were mapped to the reference genome. A correlation coefficient analysis and PCA showed that the biological replicates of *g120w* and wild type samples can be well-clustered (Figure S2).

**Figure 5:**
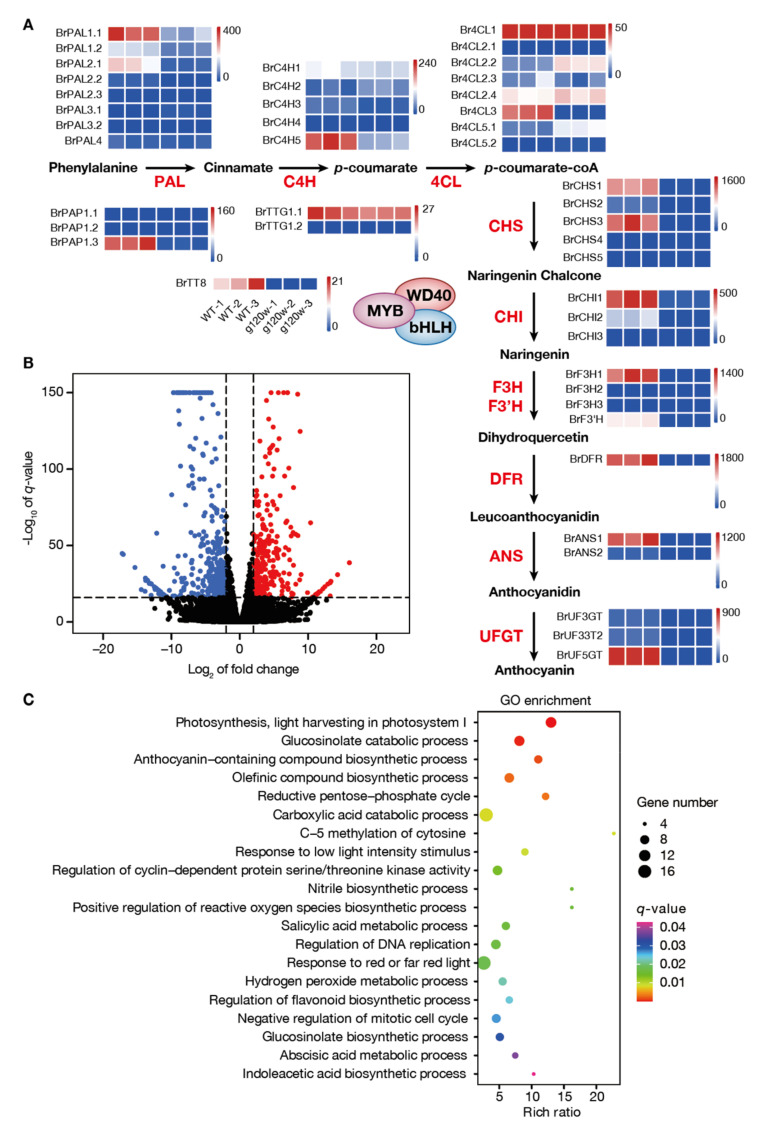
Function annotation of differentially expressed genes (DEGs) between wild type and *g120w*. (**A**) Schematic of anthocyanin biosynthetic pathway and mRNA levels of anthocyanin-related genes in wild type and *g120w* based on RNA-seq. (**B**) The volcano plot of DEGs from wild type and *g120w*. The blue spots represent downregulated DEGs, and the red spots represent upregulated DEGs. (**C**) GO enrichment analysis of DEGs between wild type and *g120w* in biological process.

For the anthocyanin-deficiency phenotype of *g120w*, candidate genes involved in anthocyanin biosynthetic pathway were screened and analyzed. Multiple copies of the anthocyanin biosynthetic genes (ABGs) were present in turnip and differentially expressed in the swollen root peel of wild type and *g120w*. The transcript levels of anthocyanin biosynthetic genes *BrPAL1.1*, *BrPAL2.1*, *BrC4H5*, *Br4CL3*, *BrCHS1*, *BrCHS3*, *BrCHI1*, *BrF3H1*, *BrDFR*, *BrANS1*, and *BrUF5GT* were much lower in *g120w* than in the wild type, and the expression of the key regulatory factors *BrPAP1.3* and *BrTT8* was also reduced (Figure 5A).

A total of 3268 genes were differentially expressed (DEGs) in the root peels of wild type and *g120w*, of which 1728 were upregulated and 1540 were downregulated in wild type (Figure 5B). To identify the BrLETM2-induced genes, we evaluated upregulated DEGs in GO enrichment analyses. Besides genes involved in anthocyanin biosynthesis, the upregulated DEGs were also enriched in response to light stimulation, positive regulation of the reactive oxygen species (ROS) biosynthesis process and hydrogen peroxide metabolism process (Figure 5C). Consistent with their differences in anthocyanin accumulation, the ROS level of swollen root peel in *g120w* was lower than in the wild type (Figure S3), indicating that BrLETM2 may regulate anthocyanin accumulation partially through the ROS signaling pathway.

### 2.6. BrLETM2 Promotes ROS Levels of Turnip Seedlings under UV-A

**Figure 6:**
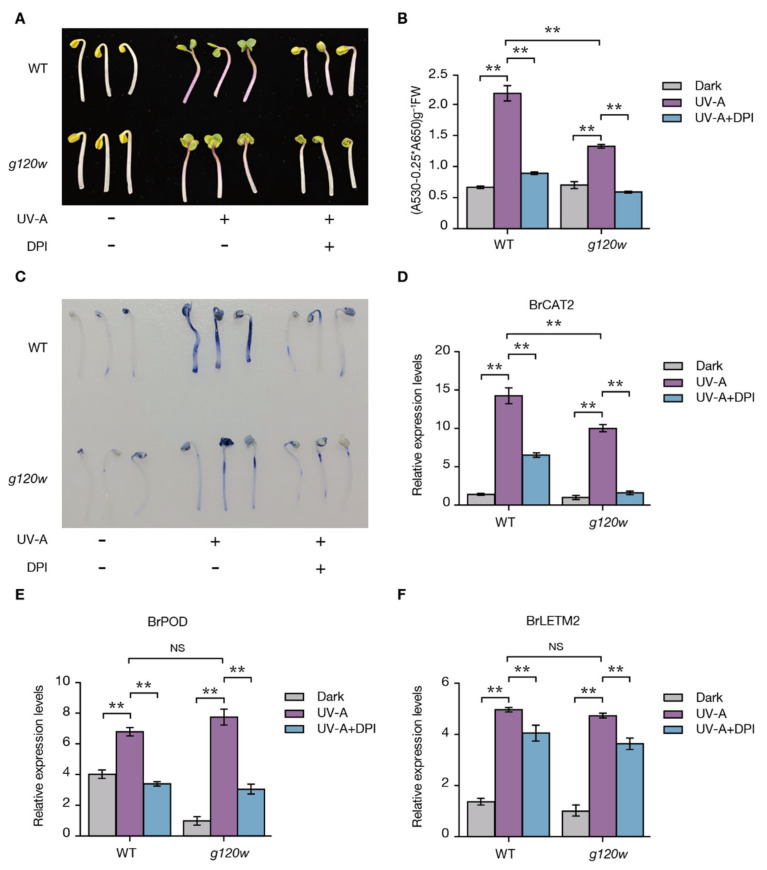
BrLETM2 is a positive regulator of ROS production and anthocyanin accumulation under UV-A irradiation. (**A**) Effects of UV-A and DPI on the morphology of turnip sprouts. (**B**) Anthocyanin levels of hypocotyls in (**A**). (**C**) NBT staining of turnip sprouts in (**A**). (**D**–**F**) qRT-PCR analysis of *BrCAT2*, *BrPOD*, and *BrLETM2* expression levels in hypocotyls in (**A**). Mean (±SD) expression levels were normalized by *BrACTIN7*, and results for *g120w* in darkness were set at 1. Asterisks indicate significant difference between different treatments in WT and *g120w* (** *p* < 0.01, Student’s *t*-test, *n* = 3).

As an inner mitochondrial membrane protein, LETM is responsible for mitochondrial Ca^2+^ levels, which affects ROS levels in cells [26,27]. ROS can promote anthocyanin accumulation in *Arabidopsis* [18]. Our previous studies showed that ROS signaling also participates in UV-A induced the anthocyanin accumulation process in swollen root peel [24]. Meanwhile, the expression of *BrLETM2* was also increased in response to UV-A irradiation (Figure S4). To validate the role of ROS in regulating the anthocyanin biosynthesis under UV-A irradiation, wild type seedlings were pretreated with diphenylene-iodonium chloride (DPI), an inhibitor of NADPH oxidase, for 2 h before exposure to UV-A. Anthocyanin levels of DPI-treated seedlings were robustly reduced after 24 h UV-A treatment compared with H_2_O-treated seedlings, indicating that ROS participates in the UV-A induced anthocyanin accumulation (Figure 6A,B). The anthocyanin level in the hypocotyl of *g120w* was significantly lower than in that of the wild type (Figure 6A,B). To elucidate whether BrLETM2 regulates ROS levels, we used nitroblue tetrazolium chloride (NBT) as a histochemical stain. Seedlings of the wild type and *g120w* were exposed to UV-A for 24 h, then the levels of superoxide (O_2_^−^) were detected by NBT (Figure 6C). The results showed that the O_2_^−^ levels in the hypocotyls of *g120w* were significantly lower than in those of the wild type. The mRNA level of the ROS-responsive genes *BrCAT2* (Catalase-2) and *BrPOD* (Peroxidase 31) were induced by UV-A and inhibited in DPI-pretreated seedlings (Figure 6D,E). Meanwhile, *BrLETM2* exhibited a similar expression pattern as ROS-responsive genes, indicating that BrLETM2 is involved in ROS production under UV-A irradiation (Figure 6F). Furthermore, *BrCAT2* was significantly decreased in *g120w* compared with that of the wild type, which is consistent with decreased O_2_^−^ levels in *g120w* seedlings. In contrast, no difference of *BrPOD* and *BrLETM2* expression level between wild type and *g120w* was observed after UV-A irradiation. These results together suggest that BrLETM2 promotes ROS production to induce anthocyanin accumulation after turnip is exposed to UV-A.

## 3. Discussion

Ultraviolet light is a key environmental signal perceived by plants that affects the flavonoid pathway and influences the levels of anthocyanins, flavonols, and proanthocyanidins [16,28,29]. As high light can induce ROS production, and ROS are also an important signal that can induce anthocyanin accumulation, studies have been conducted to identify the regulators underlying anthocyanin pigmentation mediated by ROS under solar UV radiation [30,31]. In this study, starting from the genetic analysis of the turnip mutant with anthocyanin-deficiency phenotype, we identified an LETM protein that controls the anthocyanin pigmentation of turnip swollen root peel and elucidated the mechanism of its action after UV-A exposure. We demonstrated that BrLETM2 is a positive regulator of anthocyanin pigmentation, which can be induced by UV-A and promote the ROS production. Hence, we speculate that the BrLETM2 protein modulates anthocyanin accumulation by promoting ROS production after UV-A exposure.

The *LETM* gene encodes a mitochondrial inner membrane protein, which is highly conserved across a broad range of eukaryotic organisms [32]. To date, there are only two reports about the function of LETM in plants. One showed that AtLETM1 and AtLETM2 are important proteins for the accumulation of ATP synthase in *Arabidopsis* [33]. The other showed that GhLETM1 plays a critical role in regulating stamen development and male fertility in cotton (*Gossypium hirsutum* L.) [34]. The primary functional characteristics of LETM identified in animals shows that LETM proteins act as an Ca^2+^/2H^+^ antiporter [32], and mitochondrial Ca^2+^ influx promotes ROS generation [26]. Meanwhile, because mitochondria are considered as the main source of ROS in the cell, ectopic expression of LETM1 in HeLa cells changed the membrane potential, consequently leading to increased ROS production [27]. By histochemical staining of the hypocotyl with ROS indicator, we also found that the mutation in BrLETM2 led to decreased ROS production in *g120w* (Figure 6C), which implies that LETM may work in a similar way to that in animals to enhance the ROS generation in plants.

High-intensity UV-B causes damage to DNA, proteins, and membranes in plants, but low-intensity UV-B, normal UV-A, and high-intensity visible light (usually referred to as HL) can promote ROS production and anthocyanin accumulation [15,16]. Previously, we found that genes induced by UV-A were significantly enriched for GO categories related to the ROS signaling pathway in turnip peels [24]. In the present work, after pre-treatment with DPI, the ROS level decreased under UV-A irradiation (Figure 6C), indicating that the ROS signals may integrate with the UV-A light signal pathway to modulate anthocyanin accumulation. However, we also found that the NBT staining in the hypocotyl of seedling of *g120w* was not completely lost (Figure 6C), and anthocyanin pigmentation was also not completely lost in the swollen root peels (Figure 1A,B). Considering that the photoreceptors and light signal transduction pathways are crucial for light-induced anthocyanin accumulation [9,35], it is reasonable to speculate that the LETM-mediated ROS signaling pathway only acts as a secondary messenger in this process.

The anthocyanin structural genes and regulatory genes in *B. rapa* have been identified by comparative analysis of genomic sequences of *B.rapa* with *A. thaliana* [36]. Most of the ABGs in *B. rapa* exist in multiple copies, indicating that these ABGs would likely function redundantly and determine the anthocyanin accumulation of *B.rapa* species in a spatial and temporal manner. However, the key ABGs in regulating anthocyanin biosynthesis in the peels of the swollen root of turnip have not been characterized. Based on our RNA-seq data, we identified a series of candidate genes that may contribute to anthocyanin accumulation in swollen root peel, which showed high expression levels in WT and a robust reduction in *g120w* (Figure 5A). Although other ABGs were not expressed in root peel, they may have functions in response to specific stimuli or in other organs that need to be further investigated.

MBW transcriptional regulators are generally conserved in regulating the expression of anthocyanin biosynthetic genes in eudicots [37,38]. We found that the mRNA levels of *BrLETM2* and *BrPAP1.3* genes can be induced after UV-A irradiation (Figure 6F, Figure S4), but the mRNA level of *BrPAP1.3* dramatically decreased in the *g120w* mutant (Figure 1F). The ectopic expression of *BrLETM2* induced *AtPAP1* but not *AtTT8* in *Arabidopsis* (Figure 4H,I). Xu et al. reported that ROS induce the production of anthocyanin by upregulating the LBGs due to the upregulation of *AtPAP1* and *AtTT8* in *Arabidopsis* [18]. These results indicate that BrLETM2 may work upstream of BrPAP1.3 and active expression of *BrPAP1.3* by ROS signaling to promote anthocyanin accumulation. Although the mRNA level of *BrLETM2* can be induced by UV-A, the detailed mechanism remains unknown. Whether this process is mediated by the photoreceptors or light signal transduction factors such as COP1 and HY5 needs to be confirmed with further research.

## 4. Materials and Methods

### 4.1. Plant Materials and Growth Conditions

Tsuda turnip was developed at the Institute of Flower Bioengineering in Northeast Forestry University. The wild type and *g120w* and F_2_ population of a cross between the two lines were grown in a greenhouse. Two months after sowing, when the roots had begun to swell, epidermal peels were used to measure anthocyanins and extract DNA/RNA. Turnip seedlings were grown on filter paper. For UV-A light (360 nm) treatment, etiolated seedlings were kept in darkness for two days, then transferred to UV-A (3W m^−2^) for 24 h. Hypocotyls of these seedlings were used for further analysis.

For Arabidopsis, *BrLETM2^WT^-OE* and *BrLETM2^mu^-OE* transgenic lines were derived from Col-0 ecotype. Seedlings were grown on Murashige and Skoog medium for 14 days. Mature plants were grown in a plant growth room with a 16-h light (22 to 24 °C)/8-h dark (17 to 19 °C) cycle.

### 4.2. Anthocyanin Measurement

Samples were weighed and ground to a powder in liquid nitrogen, then suspended in 1 mL of methanol containing 1% (*v*/*v*) HCl and incubated at 4 °C for 24 h. The solution was centrifuged at 14,000× *g* for 5 min. Then, 0.2 mL of each supernatant was transferred to a well in a 96-well plate, and the absorbance of samples at 530 nm (A530) and 650 nm (A650) was recorded using a microplate reader (TECAN). Anthocyanin concentrations were determined using the following equation: (A530 − 0.25 * A650)/Fresh weight (g).

### 4.3. BSA-seq Analysis and Phenotype Association Assay

Total DNA was extracted from leaves by a modified CTAB protocol. In brief, samples were ground into power by liquid nitrogen and put into 2% CTAB solution (2% CTAB, 100 mM Tris-HCL, 20 mM EDTA, 1.4 mM NaCl). Then, samples were purified by P-buffer (phenol: chloroform: isoamylol = 25:24:1) twice and precipitated by isopropanol. After centrifuging, the DNA pellet was washed by 70% ethanol and eluted by sterile water.

For two parental DNA pools, leaves from 10 wild type and 10 *g120w* mutant plants were mixed separately and used for WT and *g120w* libraries. For two F_2_ DNA pools, equal amounts of DNA from 30 PP-type and 30 WP-type F_2_ individuals were used for PP-bulk and WP-bulk libraries. BSA-seq data of four libraries were generated by paired-end sequencing with an Illumina HiSeq 2500 (Illumina Inc., San Diego, CA, USA) NGS platform. Trimmomatic software and FASTQC software were used to obtain clean reads and filter high-quality reads. Filtered clean reads from four libraries were separately mapped to the *Brassica rapa* v1.5 reference genome (http://brassicadb.org/brad/index.php (accessed on 28 December 2019)) using BWA software. All SNP variation information was generated by GATK2.7 software [39]. Informative SNPs were filtered by comparison of PP-bulk and WP-bulk pools with SAM tools for further SNP-index analysis [40]. The SNP-index of each SNP position from PP-bulk and WP-bulk was calculated; a ΔSNP-index was generated by subtraction of the WP-bulk SNP index from the PP-bulk SNP index. The ΔSNP-index across chromosomes of the *B. rapa* genome was performed by sliding-window analysis with a 1 Mb window size and 10 kb step size. The gene functions were annotated with ANNOVAR [41].

To confirm mutant loci, CAPS markers in the candidate region that may be linked with the mutated genes were screened based on SNP data from BSA-seq analysis and confirmed by co-segregation analysis. A total of 270 recessive mutant F_2_ plants were used for fine mapping. A 200~500-bp target fragment with an SNP was amplified by specific primers (Table S2). For enzyme digestion, 5 U of restriction enzyme was added into a tube that contained 8 μL PCR products, 1.5 μL 10× restriction buffer, and water, and incubated at 37 °C for 3 h. Bands were separated electrophoretically in 2~5% agarose.

### 4.4. RNA Isolation and Gene Expression Analysis

The total RNA was extracted with TRIZol-Reagent (Invitrogen, Carlsbad, CA, USA). The cDNA of samples was generated using TransScript One-Step gDNA Removal and a cDNA Synthesis SuperMix kit (TransGen Biotech, Beijing, China) according to the manufacturer’s protocol. The qRT-PCR was performed with SYBR Green PCR master mix (Roche, Rotkreuz, Switzerland). Relative expression levels were calculated by the 2^−ΔΔCt^ method. The expression of target genes was normalized using the *BrACT7* as a reference gene for turnip and *AtACT8* for Arabidopsis. All gene-specific primers used for qRT-PCR analysis are listed in Supplemental Table S2.

### 4.5. RNA-seq of Differential Gene Expression

Samples for RNA-seq were taken from the swollen root peels of wild type and *g120w* mutant grown in sunlight, with three biological replicates for each line. RNA-seq transcriptome libraries were prepared following TruSeqTM RNA sample preparation kit from Illumina, using 1μg of total RNA. Paired-end libraries were sequenced using Illumina NovaSeq 6000 sequencing by Shanghai BIOZERON Co., Ltd., Shanghai, China.

The raw paired-end reads were trimmed and quality controlled using Trimmomatic (parameters SLIDINGWINDOW: 4:15 MINLEN:75). FastQC software was used for statistics and analysis of raw data and clean data. Then, clean reads were separately aligned to the reference genome (*Brassica rapa* v1.5) with orientation mode using hisat2 software with default parameters [42].

FeatureCounts software v1.6.3 (http://subread.sourceforge.net (accessed on 10 January 2020)) and the fragments per kilobase of exon per million mapped reads (FRKM) method were used to count reads for each gene and calculate the expression level for each gene, respectively. The R statistical package edgeR (http://www.bioconductor.org/packages/release/bioc/html/edgeR.html/ (accessed on 10 January 2020)) was used for the differential expression analysis. The threshold fold-change ≥4 and FDR <0.05 were used to screen for significant DEGs. GO functional enrichment was carried out using Gene Ontology online (http://geneontology.org (accessed on 13 January 2020)) to annotate the functions of the DEGs. DEGs were considered to be significantly enriched in GO terms and metabolic pathways when their Bonferroni-corrected *p*-value was less than 0.05.

### 4.6. NBT Staining and ROS Measurement

O_2_^−^ levels were detected as previously described with minor modifications [43]. The seedlings were placed in the NBT staining solution containing 1mg mL^−1^ NBT in 50 mM sodium phosphate buffer (pH 7.5). The seedlings were then incubated for 6 h in the dark with gentle shaking. The staining solution was replaced with absolute ethanol in a boiling water bath for 30 min to remove chlorophyll. The seedlings were transferred to filter paper with 60% *v*/*v* glycerol until images were taken.

Briefly, swollen root peels were ground using a mortar and pestle in buffer (9 mL mg^−1^ fresh weight) containing 50 mM Tris-HCl (pH 7.4). The homogenate was centrifuged at 12,000 rpm for 20 min. The supernatant was transferred into a new tube for measurement. The procedure of ROS detection used was Plant Reactive Oxygen Species (ROS) ELISA Kit (MBS281870) followed the manual as described.

### 4.7. Plasmid Construction and Plant Transformation

The coding sequence (CDS) of *BrLETM2* was isolated from the cDNA of swollen root peels of wild type and *g120w* using 2× Phanta Max Master Mix (Vazyme), and was then separately cloned into pCMP1-35S vector using ClonExpress II One Step Cloning Kit (Vazyme) and individually inserted into *Agrobacterium* strain *GV3101*. Plasmids carrying 35S:BrLETM2^WT^ and 35S:BrLETM2^mu^ were inserted into *Arabidopsis thaliana* ecotype Col-0 using a general flower dip method [44]. Seedlings were screened for the transgene on Murashige and Skoog (MS) medium supplied with 40 μg/mL hygromycin, and the recombinants were confirmed by PCR amplification after they were grown in soil for two weeks.

### 4.8. Phylogenetic Analysis

BrLETM2-related proteins in plant species (Figure 3) were identified using a BLASTP search of the phytozome v12.1 database (https://phytozome.jgi.doe.gov/pz/portal.html (accessed on 23 March 2021)) and default parameters. The retrieved sequences were aligned with ClustalW in MEGAX software. The phylogenetic tree was constructed using the neighbor-joining method with default parameters besides 1000 bootstrap replications.

## Data Availability

Data are given in the article or in supplementary material.

## Supplementary Materials

Supplementary Materials can be found at https://www.mdpi.com/article/10.3390/ijms22073538/s1, Figure S1: Amino acid sequence alignment of BrLETM2 in wild type and g120w, Figure S2: Biological replication analysis of RNA-seq samples, Figure S3: ROS levels of swollen root peel in wild type and g120w, Figure S4: Anthocyanin contents and expression analysis of BrLETM2 and BrPAP1.3 in swollen root peel of wild type after 6h UV-A irradiation, Table S1: List of SNPs within candidate region for g120w mutant, Table S2: Primers used in this study.
