# Supplementary material for: BrLETM2 Protein Modulates Anthocyanin Accumulation by Promoting ROS Production in Turnip (Brassica rapa subsp. rapa)
Source: Int J Mol Sci. 2021 Mar 29;22(7):3538. doi: 10.3390/ijms22073538 (PMC8036442; doi:10.3390/ijms22073538)
Supplement: Supplementary file 1 [file ijms-22-03538-s001.pdf]

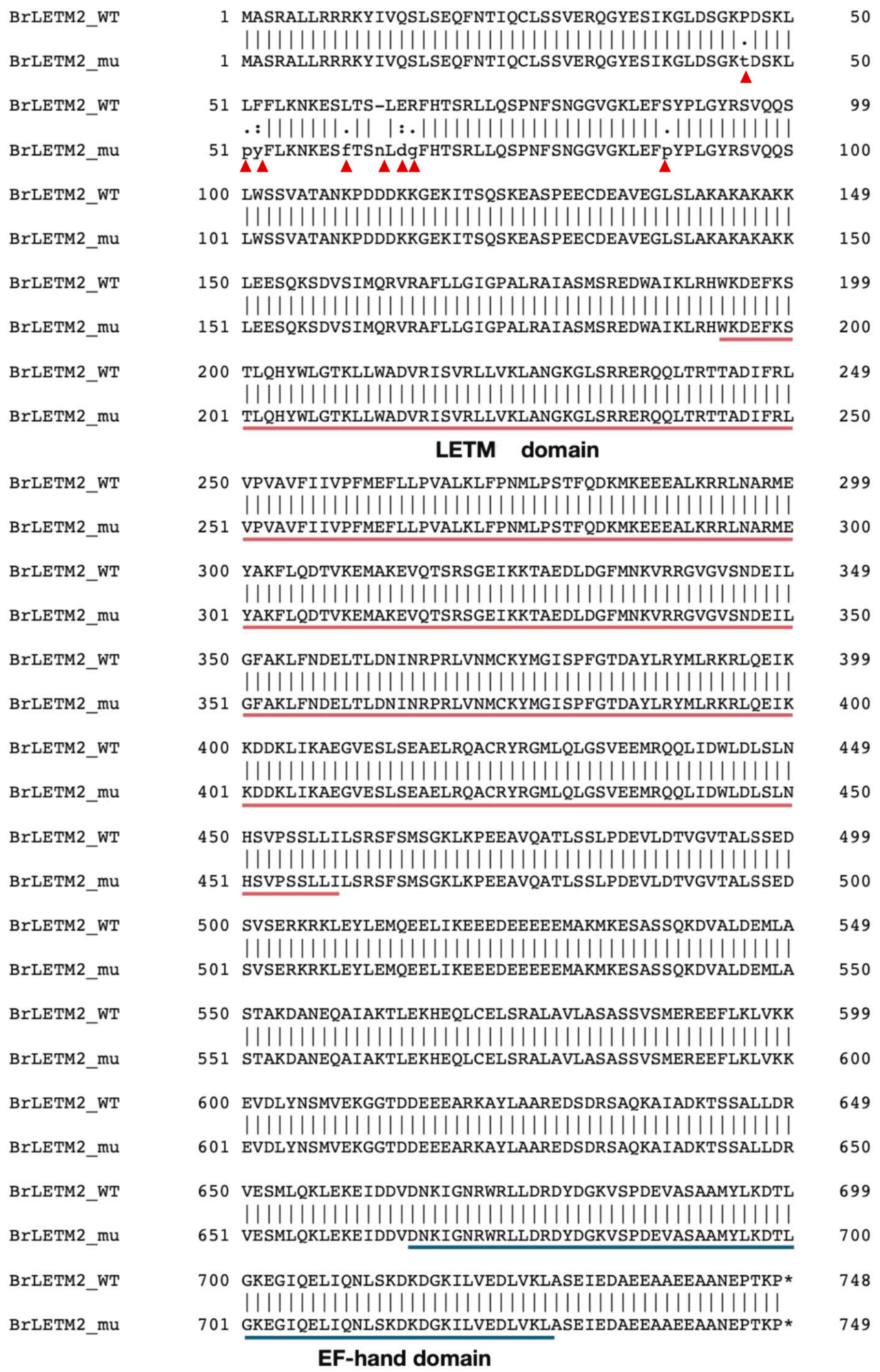

Figure S1. Amino acid sequence alignment of BrLETM2 in wild type and *g120w*. The variations of BrLETM2 in wild type and *g120w* were indicated by red triangles; Seven amino acid substitutions are P46T、L51P、F52Y、L60F、E64D、R65G and S88P, and an asparagine was inserted between S62 and L63. Red line indicates LETM domain; Blue line represents EF-hand domain.

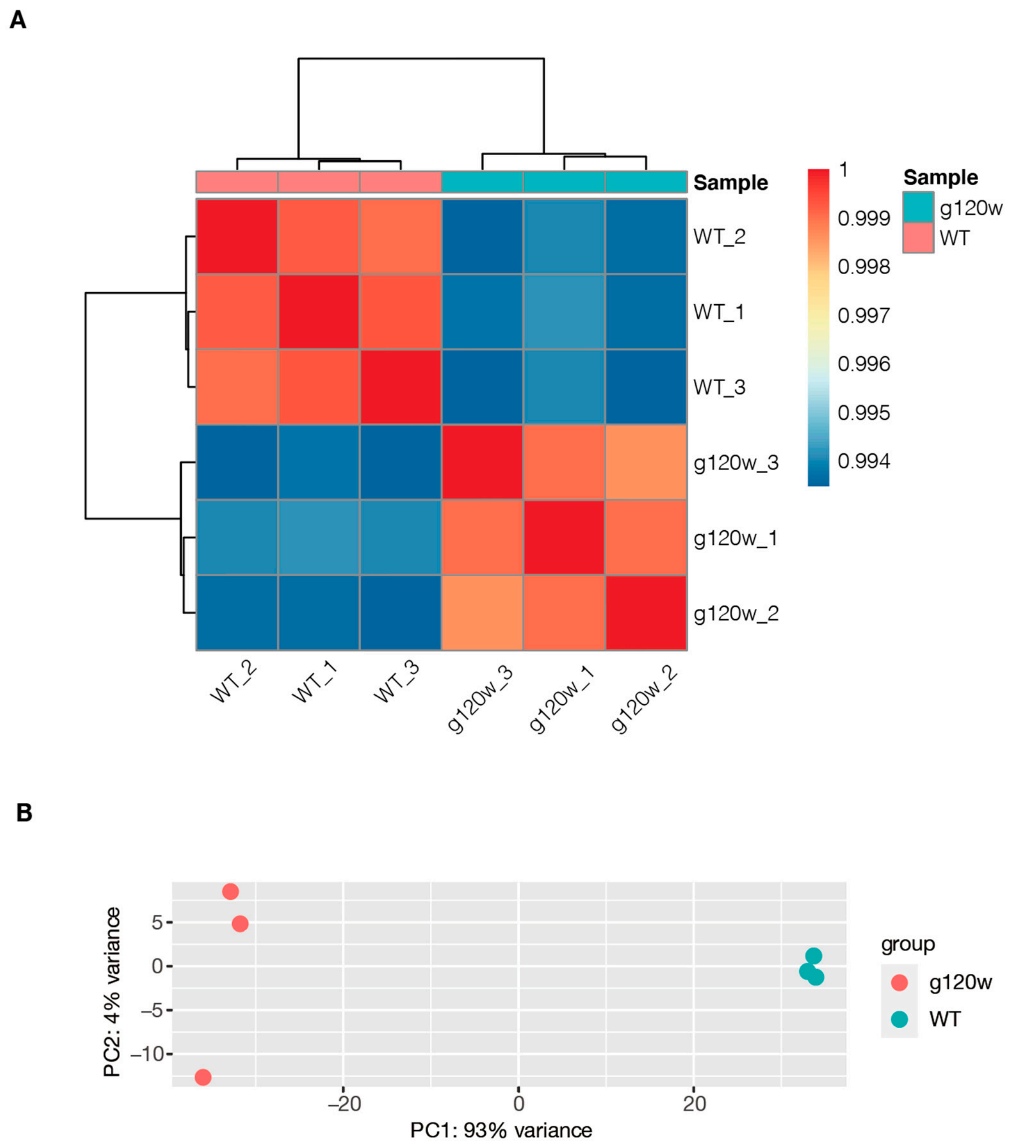

Figure S2. Biological replication analysis of RNA-seq samples. (A) Correlation coefficient by sample-based hierarchical clustering of RNA-seq data. (B) PCA analysis of biological replications samples of WT and *g120w*.

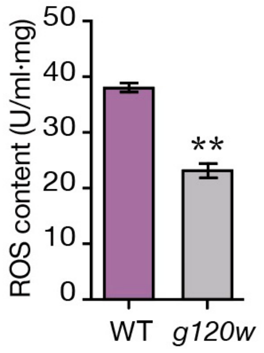

Figure S3. ROS levels of swollen root peel in wild type and *g120w*.

Asterisks indicate significant difference between dark and UV-A treatment in wild type (**$P < 0.01$, Student's t-test, n = 3).

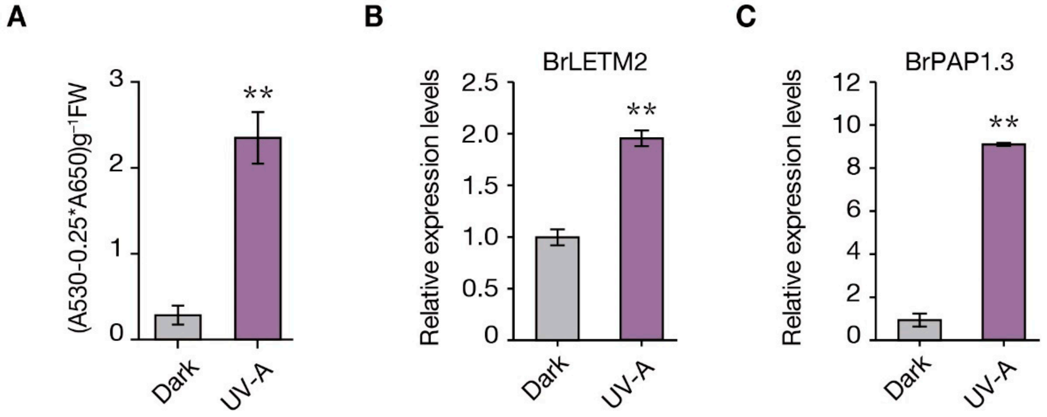

Figure S4. Anthocyanin contents and expression analysis of BrLETM2 and BrPAP1.3 in swollen root peel of wild type after 6h UV-A irradiation. (A) UV-A induces anthocyanin accumulation in swollen root peel. (B, C) qRT-PCR analysis of BrLETM2 and BrPAP1.3 expression in response to UV-A light. Asterisks indicate significant difference between dark and UV-A treatment in wild type (**$P < 0.01$, Student's t-test, n = 3).

Table S1. List of SNPs within candidate region for *g120w* mutant.

Chr	position	REF	ALT	Gene ID	Type	Annotation
7	19051951	A	G	Bra003922	nonsynonymous	pentatricopeptide (PPR) repeat-containing protein
7	19197923	T	A	Bra003947	nonsynonymous	JAZ9, TIFY7; TIFY7
7	19206730	G	A	Bra003949	nonsynonymous	protein mak16-like
7	19376237	T	A	Bra003980	nonsynonymous	ATHB13 transcription factor
7	19558785	A	G	Bra004010	nonsynonymous	DNA-directed RNA polymerase (RPOMT)
7	19561331	G	C	Bra004010	nonsynonymous	DNA-directed RNA polymerase (RPOMT)
7	19594639	A	G	Bra004014	nonsynonymous	peroxidase 11-like
7	19820391	T	A	Bra004050	nonsynonymous	AtIDD14 transcription factor
7	19934127	T	C	Bra004068	nonsynonymous	CSK (CHLOROPLAST SENSOR KINASE)
7	19935112	T	A	Bra004068	nonsynonymous	CSK (CHLOROPLAST SENSOR KINASE)
7	20221725	T	C	Bra004118	nonsynonymous	calcium-binding EF hand family protein
7	20249621	A	T	Bra004124	nonsynonymous	small nuclear ribonucleoprotein
7	20401669	C	T	Bra004157	nonsynonymous	F-box family protein
7	20613708	G	T	Bra004190	nonsynonymous	glycine-rich protein
7	20613818	C	A	Bra004190	nonsynonymous	glycine-rich protein
7	20700774	A	G	Bra004206	nonsynonymous	SWEETIE-like protein
7	21005911	T	C	Bra004270	nonsynonymous	RNA recognition motif (RRM)-containing protein
7	21435418	A	C	Bra004348	nonsynonymous	bHLH49 transcription factor

Table S2. Primers used in this study.

Primer names	Forward primer sequences (5'-3')	Reverse primer sequences (5'-3')	Purpose
CAPS4086-haeiii	ATCAGGCAGTGAATCTAGCGA	AGAATCTGAACACCTTGTTGGA	CAPS
CAPS5406-apaLI	CCGCCGTGAAGGTAGGAATC	TCAGGAAGACCGTGCGACAT	CAPS
CAPS1639-acci	TTGAAGGTGGCGAAGTAAAG	GAAGACTGGCAGCACGAGA	CAPS
CAPS9510-BstEII	GGAGACAACCCGAATCTTTAC	TAGACGCAACCACCCTCAC	CAPS
CAPS5474 TaqI	GGTGGGTTTGTGGAAGGAGG	AGGATAGCCGTTGGCAGGTTAG	CAPS
CAPS1725 HinfI	TAATTTTAGTAATGGAGGAGTTGGA	CCCTTTTTATCATCATCAGGCTTGT	dCAPS
4118-4119 indel	TTTATAAGTAGGCTACACCTCCTCTTC	GACTCGTCACTCAGCCCTGAG	indel
CAPS7234 AccI	GGTCCTTCGCTAAGCTACCAATGC	GGTCCTGGCCATGGAATCAGGTGTA	dCAPS
CAPS5241-haeiii	GGTAGCCGTGTTGTCTATTTTC	ATCACTAGCCGTCAGCGTC	CAPS
CAPS4214-SacI	CTTCATGTTGGTCACGGAAAG	ACTGGGACGACGAGGATGT	CAPS
CAPS5404-AccI	ACCTTCACCACCCATTCCC	TGCCACTGCCTCGCACTATC	CAPS
CAPS0954-Ncoi	GCATCTCCACTTTACCCCACC	TTTGAGGACTTTGTCGGCATC	CAPS
BrLETM2-CDS	GCAAAAAAAATGGCTTCAAGAGCG	CAGAAATATGAATTGTGAAAGTGAGA	Overexpression
qBrLETM2	GCGAGATTGAAGATGCTGAAG	TGAATTGTGAAAGTGAGATAGCAC	qRT-PCR
qBrCHS1	GGGACTCACCTTCCATCTCCTC	CGCGTGGCTCTCATCTTCTCT	qRT-PCR
qBrDFR	TGGTGCCAAGGGACGTTATG	TTGCCTGAGAAACTCGGAGATAG	qRT-PCR
qBrANS1	CCTCCAAGGACGTTTGCTCA	GACTTCATCCTTTTTCTCAGTTACC	qRT-PCR
qBrPAP1.3	GCCAGAAGCTGACATTGTTC	CAAACTTTCCCACCACCTATT	qRT-PCR
qBrTT8	CGACAATCATTTTGAGGCAGAG	TCAGCAATGGTTGGTTTCTTCC	qRT-PCR
qBrACT7	GCTCAGTCCAAGAGAGGTATTC	GCTCGTTGTAGAAAGTGTGATG	qRT-PCR
qBrCAT2	TAAGCTTTGTCAATCTAATCCGA	GATGACATAAATGAATCTCCATAAA	qRT-PCR
qBrPOD	TCGTTTAACAAAGCGGAACGTGAT	GTAAGGTCCTCCTACCATTGTG	qRT-PCR
qAtDFR	GCAACCATTCTTACTATCTCC	TCTCATCAACACCTTCAAAC	qRT-PCR
qAtLDOX	ACTTCTTTCATCTTGCGTATC	TCGTTGCTTCTATGTAATCAC	qRT-PCR
qAtCHS	CGAGATGAGGAGGAAGTC	GGTAGGTAGGCAGATAGAAG	qRT-PCR
qAtPAP1	AGATAAGAAGAAAGACCAACTAGTG	CCAAGGTGTCCCCCTTTTC	qRT-PCR
qAtTT8	AGAGCATCAGCAAGTGAA	GCGGTAGCCTCTTATCTT	qRT-PCR
qAtACT8	GCTCCGTATTGCTCCTGAAG	GAGGATAGCATGTGGAAGTGAG	qRT-PCR
